# The Impact of Antimicrobial Resistance and Aging in VAP Outcomes: Experience from a Large Tertiary Care Center

**DOI:** 10.1371/journal.pone.0089984

**Published:** 2014-02-27

**Authors:** Marios Arvanitis, Theodora Anagnostou, Themistoklis K. Kourkoumpetis, Panayiotis D. Ziakas, Athanasios Desalermos, Eleftherios Mylonakis

**Affiliations:** 1 Department of Medicine, Infectious Diseases Division, Rhode Island Hospital, Warren Alpert Medical School of Brown University, Providence, Rhode Island, United States of America; 2 Department of Medicine, Infectious Disease Division, Massachusetts General Hospital, Harvard Medical School, Boston, Massachusetts, United States of America; University of Florida College of Medicine, United States of America

## Abstract

**Background:**

Ventilator associated pneumonia (VAP) is a serious infection among patients in the intensive care unit (ICU).

**Methods:**

We reviewed the medical charts of all patients admitted to the adult intensive care units of the Massachusetts General Hospital that went on to develop VAP during a five year period.

**Results:**

200 patients were included in the study of which 50 (25%) were infected with a multidrug resistant pathogen. Increased age, dialysis and late onset (≥5 days from admission) VAP were associated with increased incidence of resistance. Multidrug resistant bacteria (MDRB) isolation was associated with a significant increase in median length of ICU stay (19 *vs.* 16 days, p = 0.02) and prolonged duration of mechanical ventilation (18 *vs.* 14 days, p = 0.03), but did not impact overall mortality (HR 1.12, 95% CI 0.51–2.46, p = 0.77). However, age (HR 1.04 95% CI 1.01–1.07, p = 0.003) was an independent risk factor for mortality and age ≥65 years was associated with increased incidence of methicillin-resistant *Staphylococcus aureus* (MRSA) infections (OR 2.83, 95% CI 1.27–6.32, p = 0.01).

**Conclusions:**

MDRB-related VAP is associated with prolonged ICU stay and mechanical ventilation. Interestingly, age ≥ 65 years is associated with MRSA VAP.

## Introduction

Infectious complications are amongst the dominant causes of morbidity and mortality in hospitalized patients and especially in the Intensive Care Unit (ICU) setting. Indeed, recent data estimate that health-care associated infections lead to annual costs of $9.8 billion [1]. The emergence of antimicrobial resistant pathogens further aggravates this problem rendering most of our antibacterial armory useless. To alert the scientific community on the importance of this issue, the world economic forum stated that antibiotic-resistant bacteria arguably pose the greatest risk to human health worldwide [2]. One of the most notorious among the health-care associated infections is hospital-acquired pneumonia, the most important subset of which, ventilator-associated pneumonia (VAP) accounts for 36.1% of the total annual costs associated with these diseases [1].

Currently, VAP is recognized as arguably the most important ICU-related infection with an incidence that ranges from two to sixteen cases per 1000 ventilator-days [3]. Its indubitable association with significant increases in length of ICU stay and mechanical ventilation has recently led to the widespread implementation of measures to prevent its occurrence and decrease the burden of disease [4], [5]. Importantly, VAP is often associated with strikingly high rates of multidrug resistant bacteria (MDRB), further complicating its already arduous nature [6]. Finally patients with VAP are commonly colonized in their upper respiratory tract with microbes that are not directly associated with the infection but can significantly affect it. *Candida* spp. might be an important microbe in this context [7]-[9].

## Methods

We conducted a retrospective cohort study of all consecutive non-overlapping, adult patients with VAP that received their care at the medical or surgical ICUs of the Massachusetts General Hospital (MGH) between August 2005 and November 2011. This includes a general medical ICU, a neurosciences ICU, a general surgical ICU, a cardiac-surgical ICU, a coronary care unit, a transplant and a burn ICU. The study was approved by the MGH Institutional Review Board (protocol number: 2011P001011). Due to the non-interventional and retrospective nature of the study a waiver of informed consent was granted by the Institutional Review Board. Data on demographics, previous hospitalizations, medications, and lab tests were collected through the electronic medical records. Upon study approval, two of the authors independently collected all the data and were initially blinded from the objectives of the study. All patient records were anonymized and de-identified prior to any analysis.

We identified subjects through the hospital infection control database, which listed all adult VAP patients meeting the diagnostic criteria of the Center for Disease Control and Prevention (CDC) for VAP [10]. In brief, the CDC criteria for the diagnosis of VAP require a combination of clinical symptoms, imaging results and laboratory tests that lead to the diagnosis of pneumonia acquired in the hospital and not in the community in patients intubated and mechanically ventilated for at least 48 hours. Of note, a physician’s diagnosis of pneumonia was not an acceptable criterion for VAP.

Multidrug resistant bacteria were defined as follows: 1) *Pseudomonas* spp. resistant to carbapenems or antipseudomonal penicillins and an aminoglycoside and/or a fluoroquinolone, 2) *Enterobacteriaceae* spp. resistant to carbapenems or third generation cephalosporins and an aminoglycoside and/or a fluoroquinolone and 3) *Staphylococcus aureus* resistant to oxacillin [11]. Patients with negative tracheal aspirate cultures were excluded from all data analyses related to multidrug resistance.

Elderly population was defined as people ≥65 years old [12]. We assessed severity of illness by calculating the simplified acute physiology (SAPS II) score during the first 24 hours of ICU admission. We categorized patients as surgical and medical upon admission to the ICU and we also noted any history of chronic lung disease according to the electronic medical file. We defined *Candida* colonization of the upper respiratory tract as the isolation of *Candida* species from respiratory secretions, bronchial washings, or protected airway specimens. All outcome variables were calculated defining as day 0 the day of VAP diagnosis.

### Statistical analysis

Continuous data were reported as mean (Standard Deviation, SD) or median (Interquartile Range, IQR). Group comparison was made using the Mann-Whitney non-parametric test. Count data were reported as % frequencies and compared using the Fisher’s exact test. Between-group differences were adjusted by performing a multivariable logistic regression analysis, for parameters with p<0.10 at the group analysis. Adjusted effects were reported as Odds Ratio (OR) with their 95% confidence interval. Survival analysis was performed using the Kaplan-Meier method and the log-rank p statistic was reported. All tests were two-tailed, with significance level set to <0.05. Stata v11 (College Station, TX), was used for data analysis.

## Results

### Epidemiologic characteristics of VAP

Our initial search identified 208 patients with clinically defined VAP according to the CDC criteria. Of these, 8 patients were excluded from further analysis because of non-extractable data. Among the 200 included patients, the mean (SD) age was 55.8 (18) years, 151 were males while 49 were females and the ratio between white race and all other races was 4.6:1. Interestingly the ratio between surgical and non-surgical admissions was 7:1. We also assessed various comorbidities. Specifically in our population, 21% had a history of chronic obstructive pulmonary disease (COPD), 21% had diabetes mellitus, 9% had a history of malignancy while 3% had received chemotherapy before the admission that led to VAP. The mean (SD) SAPS II score upon ICU admission was 39.3 (15.2).

We assessed several outcomes in our population. Median length of ICU stay was 18 days with an interquartile range (IQR) of 11.5–25.5 days. Median length of hospital stay was 26 days (IQR: 18-39), while the median length of mechanical ventilation (MV) was 15 days (IQR: 9-24). 47 out of the 200 evaluable patients with VAP died during their hospital admission (24%) while 40 died within 30 days of VAP diagnosis (17%).

The cause of VAP was identifiable in 169/200 patients (84.5%) and is presented in Table 1. The most common microbial causes were gram negative pathogens (46.5%), followed by gram positive bacteria (31.5%), dual gram-positive and gram-negative infections (5.5%) and fungi (0.5%) Among specific pathogens, the most common were *Staphylococcus* spp. (33%), followed by *Klebsiella* spp. (11.5%), *Enterobacter* spp. (10.5%), *Pseudomonas* spp. (10%) and *Escherichia* spp. (6%). Antimicrobial sensitivity data were available for 147 patients of whom 50 presented with an MDRB (33.3%). Interestingly, methicillin-resistant *Staphylococcus aureus* (MRSA) was isolated from 35 patients (23.8%), while extended spectrum beta lactamase (ESBL) and carbapenemase producing gram negative bacteria were isolated from 9 (6.1%) and 7 patients (4.8%), respectively.

**Table 1:** Etiologic diagnosis of VAP and resistance profiles.

Pathogen	Total number (percentage)	MDR number (percent resistant)
*Staphylococcus aureus*	66 (33%)	35 (53%)
*Klebsiella* spp.	23 (11.5%)	5 (21.7%)
*Enterobacter* spp.	21 (10.5%)	3 (14.3%)
*Pseudomonas* spp.	20 (10%)	3 (15%)
*Escherichia coli*	12 (6%)	0 (0%)
*Serratia* spp.	9 (4.5%)	0 (0%)
*Hemophilus* spp.	8 (4%)	0 (0%)
*Acinetobacter* spp.	7 (3.5%)	0 (0%)
*Stenotrophomonas maltophilia*	5 (2.5%)	5 (100%)
Other	13 (6.5%)	0 (0%)
Unknown	31 (15.5%)	N/A

MDR: multidrug resistant; N/A: not applicable; VAP: ventilator associated pneumonia.

### Risk factors for MDRB-caused VAP

Characteristics of the evaluable patients with MDRB-caused VAP (compared to the patient caused by non-MDR pathogens) are presented on Table 2. Importantly, the patients did not differ on SAPS II scores upon ICU admission, or on the type of admission. However, patients with MDRB-caused VAP were older (median age 63 *vs.* 55 years, p = 0.02), and a marginally significant higher percentage were undergoing dialysis before their most current admission that led to VAP (22% *vs.*10%, p = 0.08). Also, MDRB were more commonly isolated in patients with late VAP (≥5 days) compared to early VAP (<5 days after hospital admission) (32% *vs.*10%, p = 0.004).

**Table 2:** Characteristics of MDR vs non-MDR patients with VAP.

	MDR (n = 50)	Non-MDR (n = 97)	p-value
**Demographics**			
**Median age (IQR)**	63 (49–75)	55 (39–69)	0.02
**Female Sex**	20%	29%	0.25
**Early VAP (<5 days)**	10%	32%	0.004
**Non-surgical admission**	18%	8%	0.10
**Comorbidities**			
**Median SAPS II (IQR)**	41 (30–49)	37 (27–49)	0.58
**Cancer**	8%	11%	0.77
**Pulmonary comorbidities**	28%	16%	0.13
**Dialysis**	22%	10%	0.08
**Diabetes**	26%	15%	0.18
**ARDS**	12%	10%	0.78
**Outcomes**			
**Median ICU stay (IQR)**	19(14–30)	16 (10–23)	0.02
**Median hospital LOS (IQR)**	32 (18–42)	24 (18–34)	0.07
**Median MV duration (IQR)**	18 (11–30)	14 (8–20)	0.03
**Bacteremia**	29%	22%	0.41
**30-day mortality**	31%	21%	0.23
**Cox regression analysis for age, sex, MDR, Candida colonization in relation to 30-day mortality**			
	**Hazard Ratio**	**95% Confidence Intervals**	**p-value**
**Age**	1.04	1.01–1.07	0.003
**Male gender**	0.83	0.36–1.91	0.66
**Candida colonization**	0.42	0.14–1.23	0.12
**MDR**	1.12	0.51–2.46	0.77

ARDS: acute respiratory distress syndrome; ICU: intensive care unit; IQR: interquartile range; LOS: length of stay; MDR: multidrug resistant; MV: mechanical ventilation; VAP: ventilator associated pneumonia.

### Outcomes of MDRB-caused VAP

Interestingly, patients with MDR bacteria had significantly longer ICU stay (median of 19 *vs.*16 ICU days, p = 0.02) and median MV duration (18 *vs.*14 days, p = 0.03). However, we did not find any significant increase in 30-day mortality (31% *vs.*21%, log-rank p = 0.23) in patients with MDRB VAP compared to patients with VAP caused by non-MDRB. Furthermore, in multiadjusted analysis for age, sex, *Candida* spp. colonization, and MDRB, only the effect of age (Hazard Ratio (HR) 1.04; 95% CI 1.01–1.07, p = 0.003, per year increase) was significant (Table 2).

### VAP in the geriatric population

Based on our finding that increased age is associated increased mortality in patients with VAP, and the lack of any studies on the impact of VAP caused by MDRB in the elderly, we separated our population in two groups using the cutoff of 65 years which, although arbitrary, is widely used in studies on the geriatric population [12]. The results of this stratification are summarized in Table 3. Indeed, 30-day mortality was significantly higher in the elderly (log-rank p = 0.049). Interestingly, the two populations did not differ on disease severity on ICU admission (median SAPS II score 37.5 *vs.*37, p = 0.48). However, the older population had higher prevalence of pulmonary comorbidities (37% *vs.* 13%, p = 0.005) and marginally higher prevalence of diabetes (24% *vs.* 13%, p = 0.08).

**Table 3:** VAP patients stratified by age.

	Age ≥65 (n = 72)	Age <65 (n = 128)	p-value
**Demographics**			
Female gender	28%	23%	0.49
Non-surgical admission	10%	14%	0.51
**Comorbidities**			
Median SAPS II (IQR)	37.5 (32–48)	37 (25–50)	0.48
ARDS	17%	13%	0.70
Cancer	11%	10%	0.81
Dialysis	17%	20%	0.71
Diabetes	24%	13%	0.08
Pulmonary	31%	13%	0.005
**Cause of VAP**			
Gram–/Gram+/Both*	49%/48%/3%	60%/32%/8%	0.08
*Klebsiella* spp.	11%	15%	0.50
*Morganella* spp.	0	2%	0.54
*Pseudomonas* spp.	14%	11%	0.47
*Acinetobacter* spp.	1%	7%	0.13
*Enterobacter* spp.	11%	13%	0.81
*Stenotrophomonas maltophilia*	2%	4%	0.66
*Escherichia coli*	11%	5%	0.14
*Serratia* spp.	5%	6%	1.0
*Haemophilus* spp.	2%	7%	0.26
*Staphylococcus aureus*	48%	35%	0.11
*Enterococcus* spp.	3%	2%	0.63
Multidrug-resistant bacteria**	44%	28%	0.07
Carbapenemase producing gram negatives	4%	5%	0.62
Extended spectrum beta lactamase producing gram negatives	5%	7%	1.0
MRSA	36%	16%	0.01
**Outcomes**			
Bacteremia	19%	25%	0.37
Median LOS (range)	28.5 (5–116) d	25 (8–98) d	0.68
Median ICU stay (range)	19 (4–84)	17 (4–74)	0.17
Median MV duration (range)	14 (1–87)	15 (2–87)	0.85
Day 30 all-cause mortality	30%	15%	0.01
**Logistic regression analysis for all variables with p<0.10**			
	**Odds Ratio**	**95% Confidence Intervals**	**p-value**
MRSA	2.83	1.27–6.32	0.01
Diabetes	2.10	0.88–5.03	0.10
Pulmonary	2.51	1.07–5.88	0.03

ARDS: Acute respiratory distress syndrome; ICU: intensive care unit; IQR: interquartile range; LOS: length of stay; MRSA: Methicillin-resistant *Staphylococcus aureus*; MV: mechanical ventilation; SAPS II: simplified acute physiology score II; VAP: ventilator associated pneumonia.

*Results shown are percentages of the 167 patients for whom a microbiological diagnosis of VAP was successful.

**Results shown are percentages of the 147 patients for whom data on antimicrobial sensitivities were available.

Strikingly, when assessing the etiology of VAP in the two populations, we found that geriatric patients had marginally higher incidence of MDRB-caused VAP (44% *vs.* 28%, p = 0.07), but the etiology was significantly different and more than 1/3 of VAP in this population was caused by MRSA VAP (36% *vs.* 16%, p = 0.01). Upon multiadjusted analysis, we found that MRSA was isolated 2.83 times more commonly from VAP patients older than 65 years compared to the younger group (OR 2.83, 95% CI 1.27–6.32, p = 0.01).

### VAP and *Candida* spp. colonization

Because, as discussed below, there are reports that link colonization of the respiratory tract with *Candida* spp. with higher mortality in VAP, we evaluated the impact of *Candida* spp. in our population. We found that colonized and non-colonized patients did not differ in VAP etiology or in severity of illness based on the SAPS II score (36 *vs.* 37 respectively, p = 0.5). Specifically, among *Candida* colonized patients, 65% had Gram-negative VAP and 31% Gram-positive while among non-colonized patients 51% had Gram-negative VAP *vs*. 41% Gram-positive. When assessing the effect of *Candida* spp. colonization of the upper respiratory tract we did not find any association between *Candida* spp. and MDR VAP (23% of MDR had *Candida* spp. *vs*. 35% for non-MDR, p = 0.18). Notably, *Candida* spp. colonization was associated with increased ICU stay (18 *vs.* 13.5 days, p = 0.03), but not with prolonged MV (15 *vs.* 12.5 days, p = 0.16) or with higher 30-day mortality (19% *vs*. 26%, log-rank p = 0.14).

## Discussion

In this study, we sought to evaluate the effect of MDR organism isolation in patients with clinically defined VAP so we compared the outcomes of MDRB VAP patients with non-MDRB VAP and assessed for confounding factors. Based on the fact that MDR pathogens are more difficult to combat with traditional antimicrobial agents and on reports about the impact of MDR bacteria on the general population of hospitalized patients [13], we hypothesized that these pathogens would be associated with worse outcomes. Of note, the etiology of VAP in our study was similar to those reported in the literature, as were the rest of the epidemiologic characteristics, like median age, severity scores and comorbidities [14]–[16]. Also, the outcomes of VAP did not differ from previous reports [14]. Notably, we observed a higher rate of male patients (3:1 males to females). This is probably a reflection of the unequal distribution of male and female patients in our ICUs due to the large number of trauma patients that are predominantly male and is not different to what was previously reported in similar settings [17], [18].

Indeed, in this study we were able to show that MDRB-caused VAP isolation does lead to a significant increase in ICU stay and length of MV in patients with VAP despite the fact that MDRB and non-MDRB VAP populations did not differ in disease severity. Interestingly, we did not find a significant increase in 30-day mortality in patients with MDRB-etiology of VAP. This finding significantly contributes to the hot topic of the relationship between MDRB VAP and mortality. Specifically, a recent prospective study assessed the outcomes of ICU-acquired pneumonia in association with etiology in 217 VAP patients and 135 patients with non-ventilator associated ICU-acquired pneumonia and found that resistant organisms were associated with longer ICU stay and higher rates of microbial persistence after appropriate treatment but not with increased mortality, a conclusion that remained even after separating VAP and non-VAP patients and adjusting for confounders [19]. Similarly, Depuydt et al. assessed MDRB isolation as a potential cause of worse outcomes in 192 VAP patients and found that upon multivariate analysis increased mortality in MDR VAP patients was explained by higher comorbidities in the same population [20]. Further, three independent observational studies that focused on VAP caused by *Pseudomonas* spp. reached the conclusion that resistant *Pseudomonas* spp. were not associated with increased hospital mortality [21]–[23]. Finally, a retrospective analysis of 191 *Staphylococcus aureus* VAP cases concluded that methicillin resistance was not an independent predictor of 30-day mortality [24].

On the other hand, three studies found that resistant organisms are associated with increased mortality in VAP patients [25]–[27]. It should be noted that, two of these studies did not address the issue of potential confounders when assessing mortality of MDRB VAP [27]. Moreover, one of the two reports grouped all cases of VAP caused by *Pseudomonas* spp., irrespective of antimicrobial sensitivities, together with the MDRB VAP group when assessing for difference in outcomes, a design that further complicates the interpretation of its findings [25]. Finally, in the third retrospective study that included 193 VAP cases from a tertiary care center in Taiwan [27] the rates of microbial causes for VAP were very different from those usually reported in American hospitals [14], [16], which are similar to those that we found in our study and thus this conclusion cannot be generalized. Therefore, although no observational study can provide a definitive answer to the question, neither our results nor any existing evidence can support an association between antimicrobial resistance and mortality in the VAP population.

A very intriguing explanation for these findings would lie in the complex and recently realized relationship between resistance and virulence. The acquisition of traits that lead to antimicrobial resistance often comes with a fitness cost to the bacterial pathogen [28]. Indeed, Price et al. studied 45 cases of MRSA bacteremia prospectively and found that patients who were infected with MRSA isolates with higher vancomycin minimal inhibitory concentrations had a survival benefit over patients who were infected with more susceptible MRSA strains [29]. However, we should note that this is not true in all cases of resistant pathogens. For example, some multidrug resistant microbial strains, such as the *S. aureus* USA300 strain [30] or the *P. aeruginosa* Liverpool strain are notorious for being both extensively resistant and highly virulent and have led to serious and dangerous epidemics [28]. A more plausible explanation would be that according to the latest guidelines for the management of hospital-acquired pneumonia issued by the Infectious Disease Society of America [31], hospital acquired pneumonia should be treated empirically with broad spectrum antimicrobial agents if it is diagnosed at 5 or more days after hospital admission or if several other risk factors apply. VAP usually falls under this category as it often occurs after 5 days of admission and in high risk populations. Therefore, VAP is commonly treated empirically with broad spectrum antimicrobial agents that can successfully eradicate at least some of the MDR pathogens. Taking into consideration that the mortality of MDRB infections often stems from the delayed onset of appropriate antimicrobial therapy [32], it is possible that any effect on patient survival due to MDR prokaryotes is obscured in the case of VAP thanks to the implementation of broad spectrum therapy from disease onset.

However, such a lenient policy for early broad antimicrobial coverage doesn’t come free of costs. Anti-infective agents are often associated with severe side effects especially in populations with multiple comorbidities such as patients at high risk for MDRB-VAP. Therefore, every benefit from the implementation of broad spectrum treatment strategies with multiple antimicrobials in high risk patients should be weighed against the potential for therapeutic adverse events in the same population. Nevertheless, since the lack of significant impact of MDRB VAP on mortality could be masked by the current therapeutic protocols it would be wrong to completely underestimate its importance. Indeed, we found that MDR pathogens are associated with increase in ICU stay and MV duration both of which are serious causes of morbidity. Moreover, in agreement with previous reports [33], [34], we showed that late onset VAP is associated with a significantly higher risk of MDR infection, which corroborates the recommendation of the latest treatment guidelines. Consequently, on the face of these findings, we believe that randomized trials that compare current treatment strategies with a more cautious approach that starts with narrow spectrum antimicrobials are imperative especially in the high risk groups and should be performed before any changes in the current recommendations are implemented.

Based on the significant impact of age on mortality in VAP, we also assessed the etiologic diagnosis of VAP in patients ≥65 years old. Notably, we found that the probability of MRSA VAP is almost tripled in the population, an effect that is independent of other comorbidities as proven by multivariable logistic regression analysis. Surprisingly, the significance of VAP in this population has not been studied in detail. We were able to find only two recent studies that evaluated the association between MRSA VAP and age which found that age is significantly associated with a higher risk for MRSA [24], [35]. Our study goes even further by showing that 36% of the elderly patients with microbiologically defined VAP are infected with MRSA, thus revealing the magnitude of the problem. This result is most likely associated with the living conditions of the elderly population or with their more frequent hospitalization which is associated with an increased risk of MRSA colonization [36] rather than with age itself. However, given the strikingly high prevalence of MRSA in the elderly VAP patients, this association should be seriously taken into consideration when treating geriatric people with VAP.

Because previous reports indicated that *Candida* spp. colonization of the upper respiratory tract is associated with increased risk of MDRB isolation and worse outcomes in VAP patients [11], [37], we evaluated this potential confounding factor in detail. We found that, in our population, colonization of the upper respiratory tract by *Candida* spp. is an important predictor of morbidity as it significantly prolongs ICU stay. Notably, we did not find any relationship between *Candida* and increased mortality in VAP. To our knowledge, this is the first study that evaluated the impact of *Candida* spp. colonization in a consecutive population of patients with clinically defined VAP. Two recent studies that found an association between *Candida* spp. colonization and mortality were focusing in a subset of the VAP population that did not have an identified bacterial pathogen isolated from their tracheal cultures [9], [38]. Another study that found increased mortality in colonized patients with suspected VAP excluded all patients that were colonized or infected with MRSA or *Pseudomonas* spp. thus limiting the generalization of the results in the total VAP population [37]. Moreover, in agreement with our findings, an earlier report that studied the effect of *Candida* spp. colonization on outcomes in immunocompetent individuals with mechanical ventilation found that *Candida* spp. were associated with a higher ICU stay but not with higher mortality [7]. Finally, based on a previous study that found an association between *Candida* spp. colonization and MDR in patients with suspected VAP [11], we also assessed whether the prolonged ICU stay that we found was confounded by a higher rate of MDR in our population but we did not find such a relationship. Therefore, based on our findings, *Candida* spp. should be evaluated as an independent factor that might be associated with higher morbidity but not mortality in patients with clinically defined VAP.

Limitations of our study include its retrospective design which precludes any discussion on causative relationships between exposures and outcomes. Indisputable cause and effect relationships can only be proven with interventional studies, which are often non-feasible in the case of MDR infections [39]. Therefore, data from observational studies indicating associations could be particularly useful in clinical decision making in such cases. Also, due to missing data from the electronic medical records, we had a relatively high number of cases with unknown antimicrobial sensitivities. Finally, we should note that to eliminate subjectivity in reporting cases of VAP, the CDC has very recently issued new criteria for defining and reporting ventilator-associated events [40]. Although it will take some time until all reporting and surveillance systems of hospitals have shifted toward the new definitions, it would be particularly interesting to investigate how this new effort would impact our findings and this should be the target of future studies in the field.

In conclusion, our study provides evidence that MDRB isolation is associated with increased morbidity but not mortality in patients with VAP. Also, late onset (≥5 days from admission) VAP is associated with a significantly higher rate of multidrug resistance. Interestingly, age is an independent predictor of mortality in VAP and geriatric patients have an almost threefold increased risk for MRSA VAP. These findings should be further confirmed in future multicenter trials.
